# Mid term results after bone marrow laser revascularization for treating refractory angina

**DOI:** 10.1186/1471-2261-10-42

**Published:** 2010-09-17

**Authors:** Guillermo Reyes, Keith B Allen, Pablo Álvarez, Adrian Alegre, Beatriz Aguado, MariaJose Olivera, Paloma Caballero, JoseLuis Rodríguez, Juan Duarte

**Affiliations:** 1 Department of Cardiovascular Surgery, Hospital Universitario La Princesa, c/Diego de Leon 62, Madrid 28006, Spain; 2 Department of Cardiothoracic Surgery, Mid America Heart Institute, St Luke 's Hospital, Kansas City, Missouri, USA; 3 Department of Haematology, Hospital Universitario La Princesa, c/Diego de Leon 62, Madrid 28006, Spain; 4 Department of Radiology, Hospital Universitario La Princesa, c/Diego de Leon 62, Madrid 28006, Spain; 5 Department of Nuclear Medicine, Hospital Universitario La Princesa, c/Diego de Leon 62, Madrid 28006, Spain

## Abstract

**Background:**

To evaluate the midterm results of patients with angina and diffuse coronary artery disease treated with transmyocardial revascularization in combination with autologous stem cell therapy.

**Methods:**

Nineteen patients with diffuse coronary artery disease and medically refractory class III/IV angina were evaluated between June 2007 and December 2009 for sole therapy TMR combined with intramyocardial injection of concentrated stem cells. At the time of surgery, autologous bone marrow (120cc) was aspirated from the iliac crest. A cardiac MRI and an isotopic test were performed before and after the procedure. Follow-up was performed by personal interview.

**Results:**

There were no perioperative adverse events including no arrhythmias. Mean number of laser channels was 20 and the mean total number of intramyocardially injected cells per milliliter were: total mononuclear cells(83.6 × 10^6^), CD34+ cells(0.6 × 10^6^), and CD133+ cells(0.34 × 10^6^). At 12 months mean follow-up average angina class was significantly improved (3.4 ± 0.5 vs 1.4 ± 0.6; p = 0.004). In addition, monthly cardiovascular medication usage was significantly decreased (348 ± 118 vs. 201 ± 92; p = 0.001). At six months follow up there was a reduction in the number of cardiac hospital readmissions (2.9 ± 2.3 vs. 0.5 ± 0.8; p < 0.001). MRI showed no alterations regarding LV volumes and a 3% improvement regarding ejection fraction.

**Conclusions:**

The stem cell isolator efficiently concentrated autologous bone marrow derived stem cells while the TMR/stem cell combination delivery device worked uneventfully. An improvement in clinical status was noticed in the midterm follow-up. Images test showed no morphological alterations in the left ventricle after the procedure.

## Background

Coronary artery disease (CAD) remains a leading cause of death and disability and results in a significant social and economic burden to the health care system. Currently available options for treating CAD include life style changes in conjunction with drug therapy, percutaneous coronary intervention (PCI) and coronary artery bypass graft (CABG) surgery. It is estimated, however, that 1-3% of patients presenting with diffuse CAD are not candidates for conventional revascularization and that 15-25% of patients undergoing CABG will have one or more major target areas incompletely revascularized due to diffuse coronary artery disease[1].

Incomplete revascularization is increasingly recognized as an independent predictor of operative mortality[2], particularly in the elderly[3]. Transmyocardial revascularization (TMR) is an approved surgical procedure to treat patients with diffuse coronary artery disease in which 1 mm transmural laser channels are created in ischemic myocardium which cannot be conventionally revascularized. TMR can be performed either as a stand alone procedure (sole therapy) in patients with medically refractory angina who are not candidates for conventional revascularization or in conjunction with CABG in patients who would be incompletely revascularized by CABG alone. Although sole therapy TMR has demonstrated superiority over continued medical therapy in randomized trials[3-8], its effectiveness at angina relief is not 100%. Approximately 25% of patients treated with sole therapy TMR do not experience a two class reduction in angina at one year[3,5].

Angiogenic up regulation of injured myocytes by the laser is hypothesized to provide a 'fertile' area for an enhanced stem cell paracrine effect. To increase the angiogenic response and associated clinical efficacy of TMR, the potential synergy of combining TMR with a cell-based therapy was investigated. Recently we described our Bone Marrow Laser Revascularization (BMLR) technique[9] in which a single device is used to perform holmium:YAG TMR (PHOENIX™, CardioGenesis, Irvine, CA) and inject concentrated autologous bone marrow derived stem cells. Now we describe our mid term results regarding clinical status, cardiac events and images findings in patients treated with the BMLR technique.

## Methods

### Patient Selection

Between June 2007 and December 2009, nineteen consecutive patients with diffuse coronary artery disease and with maximal refractory medical treatment class III/IV angina who were not candidates for PCI or CABG were prospectively evaluated for Bone Marrow Laser Revascularization (BMLR). Agreement by a cardiologist and two cardiac surgeons on inoperability was required for inclusion in this study. The Ethics Committee and Institutional Review Board approved this prospective, single arm open label study and informed consent was obtained from each patient. Inclusion criteria included age over 18 and left ventricle ejection fraction >25% with documented left ventricular reversible ischemia documented by echocardiography, magnetic resonance imagining or nuclear tests. Exclusion criteria included severe chronic obstructive pulmonary disease, unstable angina requiring intravenous nitrates, myocardial infarction within two weeks of surgery, decompensated congestive heart failure, refractory arrhythmias and bleeding disorders.

### Surgical Technique

Our technique has been previously described[9]. Briefly, after the bone marrow aspiration the patient was repositioned supine with the left shoulder elevated and a limited anterior lateral left thoracotomy incision was performed on a beating heart through the fifth interspace allowing exposure of the distal two-thirds of the left ventricle for BMLR. No heparinization was required. BMLR was performed using the PHOENIX combination delivery system consisting of a 1 mm flexible optical fiber (connected to a 20 watt pulsed holmium:yttrium-aluminum-garnet (holmium:YAG) laser) along with a needle injection system. A bolus of 150 mg of amiodarone was administered before starting the BMLR procedure. An average of 20 laser channels (range 15-25) were created in each patient. The three retractable needles deployed after each channel and 1 mL of concentrated mononuclear cells were injected into the myocardium around the channel.

All adverse events were recorded. Myocardial injury was monitored postoperatively measuring creatinine kinase (CKMB) and troponin. Efficacy endpoints included blinded angina assessment, change in cardiac medication usage and rates of cardiac hospital readmission following the procedure.

#### Follow-up

All patients were reviewed by a cardiac surgeon and by a cardiologist. MRI and SPECT test were performed before and 6-12 months after the procedure.

### Statistics

Patient demographics and perioperative variables were collected prospectively. Continuous variables are expressed as mean ± standard deviation and categorical data as proportions. Student's T-test or the Mann-Whitney U-test was used for continuous variables comparisons. Categorical variables were compared using chi-square analysis. Patient follow-up was performed by citation in hospital or by telephone. All statistical analyses were performed using SPSS statistical package 14.0 (SPSS Corp., Birmingham, AL, USA)

## Results

### Patient Characteristics

Nineteen patients (16 male: 3 female) with a mean age of 65.2 ± 6.1 years (range 52-78) underwent BMLR. Baseline clinical characteristics are described in Table 1. Baseline Canadian Cardiovascular Class was Class IV in seven patients and Class III in twelve patients. Fifteen patients had prior PCI procedures (mean 3.3 interventions; range: 1-7). Eight patients had previous CABG procedures. Mean baseline ejection fraction was 54% (range 30-65).

**Table 1:** Baseline clinical characteristics of patients (n = 19).

	n (%)
**Hypertension**	18 (94.7%)
**Diabetes**	11 (57.9%)
**Dyslipemia**	16 (84.2%)
**Current smoking**	4 (21%)
**Peripheral vascular disease**	4 (21%)
**Lung disease**	2 (10.5%)
**Crhonic myocardial infarction**	12 (63,2%)
**Sinus rhythm**	19 (100%)

### Safety Analyses

**Figure 1:**
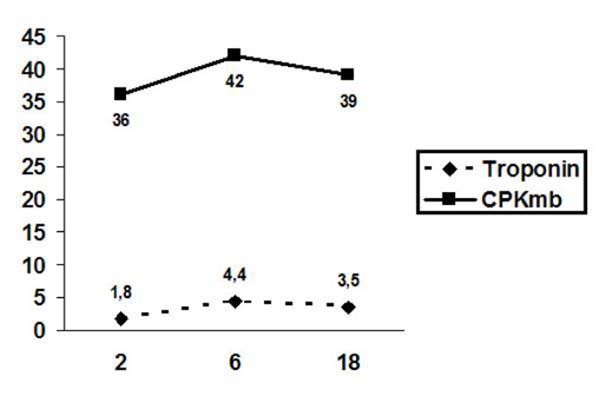
**Troponin and CPKmb levels measured at 2, 6 and 18 hours after BMLR**.

All nineteen patients enrolled in the trial underwent successful BMLR without complications including no surgical mortality and no perioperative arrhythmias. There were no complications related to the bone marrow aspiration. One diabetic patient developed a superficial wound infection in her inframammary incision three weeks following surgery which healed uneventfully. Cardiac enzymes were measured at 2, 6, and 18 hours following the procedure (Figure 1). Postoperatively median length of stay in the intensive care unit was one day and average total length of stay was 5.5 days.

Average follow-up was nineteen months (range: 2-30 months). There was one late death 28 months post procedure in a uncontrolled diabetic female due to heart failure

### Efficacy Analyses

Average follow-up was nineteen months and it was complete in all nineteen patients. Average angina class was significantly improved from baseline to follow up (3.4 ± 0.5 vs 1.7 ± 0.9; p < 0.001). All patients experienced a two class reduction in angina with 50% (7/14) angina free six months post procedure. Monthly cardiac medication usage overall was significantly decreased (348 ± 118 vs. 201 ± 92; p = 0.001) with the number of sublingual nitrates taken per month reduced (22.1 ± 30.4 vs. 1.4 ± 3.9; p < 0.001). Significant reduction in cardiac related hospitalizations in the six months following treatment with BMLR compared to the six months prior to study enrollment (0.5 ± 0.8 vs 2.9 ± 2.3; p < 0.001)

Three patients received follow on cardiac catheterization post BMLR due to advancing disease. Two were successfully intervened in territories with new lesions, the other could not receive further intervention.

### Cell Counts

Mononuclear cells (MNCs) from the bone marrow aspirate were harvested and concentrated intraoperatively. Average time to complete the bone marrow aspiration and concentrate the cells was 30 minutes. Table 2 demonstrates the mean number of total MNCs, CD34+ cells, and CD133+ cells before and after the centrifugation.

**Table 2:** Bone marrow aspiration cell counts per milliliter before and after concentration (p < 0.05 in all cases).

Bone Marrow Aspiration	**Average Total MNC (10**^**6 **^**/ml ± range)**	**Average CD34+ (10**^**6 **^**/ml ± range)**	**Average CD133+ (10**^**6 **^**/ml ± range)**
Pre Concentrate (120cc)	27.9 (15.1 - 45.0)	0.16 (0.04 - 0.3)	0.11 (0.001 - 0.2)

Post Concentrate (20cc)	81.3 (43.7 - 156.8)	0.6 (0.1 - 1.4)	0.37 (0.001 - 0.1)

### Diagnostic Results

Follow up MRI was performed 6 months post procedure. Average EF% at baseline was of 47% and average EF% at follow-up was 50%. This is a 3% increase in EF% after BMLR treatment. Dimensional measures of the left ventricle were also documented, with no change in end diastolic or end systolic volumes. Patients diagnosed using SPECT did not show changes in fixed or variable defects.

## Discussion

We combined TMR with autologous bone marrow derived stem cells injection in patients suffering angina despite medical treatment and with diffuse coronary disease non-manageable with standard surgical options. The procedure was feasible with no complications and it resulted a clinical improvement in patients. There was significant reduction in angina class, number of cardiovascular medication and cardiac related hospital admissions.

Transmyocardial revascularization is an approved surgical procedure to treat 'no option' patients with diffuse coronary artery disease. In prospective randomized trials sole therapy TMR has demonstrated a significant improvement in angina and event free survival and a reduction in cardiac related hospitalizations compared to patients randomized to maximum medical therapy alone[3,5-8]. Long term follow up of TMR as a primary therapy shows an enduring benefit over time[4,10] and 5 year follow up of one prospective randomized trial involving the sickest Canadian Cardiovascular Class IV patients has shown improved survival in the TMR treated patients[4]. In a recent meta-analysis the superiority of TMR versus maximal medical management at one and 3-5 year follow-up with regard to two class angina improvement has been confirmed[11]. The Society of Thoracic Surgery and the International Society of Minimally Invasive Cardiothoracic Surgeons (ISMICS) have published practice guidelines giving sole therapy TMR a Grade I recommendation with Level A evidence.

Although TMR's superiority over medical therapy has been demonstrated in randomized trials, in up to 25% of patients treated with TMR, angina relief is not significantly improved at one year and the percentage of patients who become angina free is approximately 20%[3,5]. In prospective series all patients significantly improve in angina class through the period of follow up.

As a potential alternative to TMR, exogenously administered biologic substances such as growth factors and stem cells have been evaluated for the treatment of medically-refractory angina. Direct intramyocardial injection of specific growth factors, such as vascular endothelial growth factor and basis fibroblastic growth factor have yielded angina improvement in inoperable patients and may positively effect left ventricular function[12]. The use of intramyocardial injection of autologous bone marrow derived mononuclear cells such as CD34+ and AC133+ stem cells has also yielded positive efficacy signals with regard to angina improvement and myocardial perfusion in patients with refractory ischemia[13,14].

TMR results in an up regulation of vascular endothelial growth factor messenger RNA and an increased expression of other angiogenic growth factors[15]. Hugh's and colleagues[16] examined the neovascularization response six months post-TMR in an ischemic porcine model and reported significant increases in vascular density in lased regions. In addition they reported that laser systems which create an injury (carbon dioxide and Holium:YAG) improved myocardial blood flow and contractile reserve in lased regions, whereas improvements were not observed following a sham thoracotomy or using a non injury producing lasers (eximer). When mechanical TMR has been compared to laser TMR, mechanical TMR resulted in no angiogenic response suggesting that a threshold injury to an ischemic myocardial region was needed to induce angiogenesis[17]. In an evaluation of their cumulative studies, Hughes and Lowe concluded that TMR induced neovascularization in lased regions is likely due to an up regulation of the angiogenic cascade secondary to an inflammatory response after laser treatment. Bone Marrow Laser Revascularization (BMLR) describes the delivery of autologous bone marrow concentrate in conjunction with TMR channels into targeted ischemic tissue. It is the hypothesized that the delivery of bone marrow derived stem cells into the order zone surrounding the channels will significantly enhance the angiogenic response compared to TMR alone. We have proved that this surgical approach is safe, easily reproducible and it can be performed in 60-90 minutes. We had no device related complications, and only one procedurally related complication - a surgical wound healing complication in a severely diabetic patient. This despite a relatively high risk patient series with diffuse coronary heart disease and most with additional cardiovascular risk factors. The procedure was performed in an average of 90 minutes with no significant operative events. It is the hypothesized that the delivery of bone marrow derived stem cells into the border zone surrounding the channels may significantly enhance the angiogenic response and resulting clinical effect compared to TMR alone.

Utilizing TMR as a biomechanical trigger to enhance the angiogenic cascade when combined with an adjunctive biological therapy is supported by enhanced perfusion and improved mechanical function when evaluated in ischemic animal models[18]. Recent animal studies provide insight into possible mechanisms of synergy between TMR and biologic substances. Atluri and colleagues[19] demonstrated that the localized acute healing response to the laser injury includes an up regulation of injured myocytes, platelet activation with growth factor release from the thrombus that forms within the laser channel, as well as the recruitment of intrinsic myocardial stem cells. In addition, Patel and colleagues[20] demonstrated enhanced stem cell retention when stem cells are injected into the border zone of a laser channel suggesting the microenvironment created by the laser-tissue interaction may be important for stem cell retention in ischemic tissue. Finally, the small, early clinical experience with TMR combined with stem cell therapy has demonstrated its safety and feasibility and the potential for improving outcomes[21,22]. Wehberg and colleagues recently demonstrated superior angina relief and significant ejection fraction improvement when sole therapy TMR was combined with platelet rich plasma compared to TMR alone. A randomize trial comparing TMR versus BMLR is required to validate this hypothesis.

This patient series demonstrated that the clinical benefit was achieved without adverse events. The diagnostics performed showed no change in the functional performance of the left ventricle. These data support the safety of and feasibility of the BMLR technique. Although special methods as three dimensional microvascular lectin angiogram [19] or the modified Clark electrode[23] has been used in animals models it is believed that conventional methods may not be sensitive enough to identify subtle changes after stem cell injection[23]. The objective of this study was to evaluate the safety and feasibility of the BMLR treatment while initially collecting effeicacy outcomes data. The MRI and SPECT performed ruled out any adverse remodeling of the left ventricle, but may not be ideal for identifying the physiologic effect of the treatment.

Our preliminary results demonstrate the safety and feasibility of combining TMR and the implantation of autologous concentrated bone marrow derived stem cells, and delivering them through a single device. In this prospective series, the magnitude of angina relief demonstrated with BMLR was favorable when compared to publish TMR as a stand-alone therapy. In addition, the bone marrow concentration method (SmartPrep2, Harvest Technologies, Plymouth, MA, USA) allowed rapid and efficient concentration of bone marrow aspirate in the operating room while obtaining high stem cell counts with minimal manipulation of the autologous material. In a recent meta-analysis[24] the mean number of mononuclear cells concentrated using cumbersome and time consuming chemical or filtering techniques was 80 × 10^6^/ml compared to 81.3 × 10^6^/ml observed with this technique. Our mean count of mononuclear cells was 81.3 (43.7 - 156.8) × 10^6 ^per millilitre and we obtained 20 cc of concentrated bone marrow mononuclear cells which may be adequate to achieve the desired clinical result.

### Limitations of the study

This is prospective single arm open label study in 19 patients to assess the safety and feasibility of the BMLR treatment. Longer follow up and randomized groups are required to assess the potential synergy of TMR combined with bone marrow derived stem cells. Cardiac imaging to evaluate perfusion and function (cardiac MRI and SPECT nuclear study) were included in this study. Additional diagnostic techniques or methods may be required to asses the physiological impact of the BMLR treatment.

A randomized trial comparing TMR versus BMLR is required to assess the superiority of adding stem cells to the TMR procedure.

## Conclusions

Cardiac surgeons are increasingly faced with a more complex patient who has developed a pattern of diffuse coronary artery disease who cannot be completely revascularized by conventional techniques. The point of care bone marrow aspirate centrifuge system provides a straight forward method for intraoperative harvesting and preparation of autologous stem cells. The combination delivery system provides for the efficient delivery of TMR and concentrated cells in targeted ischemic myocardium. This advanced treatment for inoperable CAD is safe and feasible. Prospective, randomized, multi-center trials are required to determine the degree of synergistic effect.

## Competing interests

The authors declare that they have no competing interests.

## Authors' contributions

GR designed and conducted the study drafted the manuscript. KB contributed to the study design and manuscript revision. PA was responsible for acquisition of data. AA and BA designed the hematology portion of the study design and manuscript. MJO and PC performed and analyzed the MRI tests. JLR performed and analyzed the SPECT tests. JD reviewed the final version of the manuscript. All authors read and approved the final manuscript.

## Pre-publication history

The pre-publication history for this paper can be accessed here:

http://www.biomedcentral.com/1471-2261/10/42/prepub

## References

[B1] Weintraub WS Jones EL Craver JM Guyton RA Frequency of repeat coronary bypass or coronary angioplasty after coronary artery bypass surgery using saphenous venous grafts Am J Cardiol 1994 73 2 103 12 10.1016/0002-9149(94)90198-8 8296729

[B2] Graham MM Chambers RJ Davies RF Angiographic quantification of diffuse coronary artery disease: reliability and prognostic value for bypass operations J Thorac Cardiovasc Surg 1999 118 4 618 27 10.1016/S0022-5223(99)70006-1 10504625

[B3] Allen KB Dowling RD Fudge TL Schoettle GP Selinger SL Gangahar DM Angell WW Petracek MR Shaar CJ O'Neill WW Comparison of transmyocardial revascularization with medical therapy in patients with refractory angina N Engl J Med 1999 341 14 1029 36 10.1056/NEJM199909303411403 10502592

[B4] Allen KB Dowling RD Angell WW Gangahar DM Fudge TL Richenbacher W Selinger SL Petracek MR Murphy D Transmyocardial revascularization: 5-year follow-up of a prospective, randomized multicenter trial Ann Thorac Surg 2004 77 4 1228 34 10.1016/j.athoracsur.2004.01.008 15063241

[B5] Frazier OH March RJ Horvath KA Transmyocardial revascularization with a carbon dioxide laser in patients with end-stage coronary artery disease N Engl J Med 1999 341 14 1021 8 10.1056/NEJM199909303411402 10502591

[B6] Burkhoff D Schmidt S Schulman SP Myers J Resar J Becker LC Weiss J Jones JW Transmyocardial laser revascularisation compared with continued medical therapy for treatment of refractory angina pectoris: a prospective randomised trial. ATLANTIC Investigators. Angina Treatments-Lasers and Normal Therapies in Comparison Lancet 1999 354 9182 885 90 10.1016/S0140-6736(99)08113-1 10489946

[B7] Schofield PM Sharples LD Caine N Burns S Tait S Wistow T Buxton M Wallwork J Transmyocardial laser revascularisation in patients with refractory angina: a randomised controlled trial Lancet 1999 353 9152 519 24 10.1016/S0140-6736(98)11478-2 10028979

[B8] Aaberge L Nordstrand K Dragsund M Saatvedt K Endresen K Golf S Geiran O Abdelnoor M Forfang K Transmyocardial revascularization with CO2 laser in patients with refractory angina pectoris. Clinical results from the Norwegian randomized trial J Am Coll Cardiol 2000 35 5 1170 7 10.1016/S0735-1097(00)00519-2 10758957

[B9] Reyes G Allen KB Aguado B Duarte J Bone marrow laser revascularisation for treating refractory angina due to diffuse coronary heart disease Eur J Cardiothorac Surg 2009 36 1 192 4 Epub 2009 Apr 25 10.1016/j.ejcts.2009.03.022 19394846

[B10] Aaberge L Rootwelt K Blomhoff S Saatvedt K Abdelnoor M Forfang K Continued symptomatic improvement three to five years after transmyocardial revascularization with CO(2) laser: a late clinical follow-up of the Norwegian Randomized trial with transmyocardial revascularization J Am Coll Cardiol 2002 39 10 1588 93 10.1016/S0735-1097(02)01828-4 12020484

[B11] Cheng D Diegeler A Allen K Weisel R Lutter G Sartori M Transmyocardial Laser Revascularization: A Meta-Analysis and Systematic Review of Controlled Trials Innovations 2006 1 295 313 10.1097/IMI.0b013e31802fe0a2 22436830

[B12] Losordo DW Vale PR Symes JF Dunnington CH Esakof DD Maysky M Ashare AB Lathi K Isner JM Gene therapy for myocardial angiogenesis: initial clinical results with direct myocardial injection of phVEGF165 as sole therapy for myocardial ischemia Circulation 1998 98 25 2800 4 9860779 10.1161/01.cir.98.25.2800

[B13] Losordo DW Schatz RA White CJ Udelson JE Veereshwarayya V Durgin M Poh KK Weinstein R Kearney M Chaudhry M Burg A Eaton L Heyd L Thorne T Shturman L Hoffmeister P Story K Zak V Dowling D Traverse JH Olson RE Flanagan J Sodano D Murayama T Kawamoto A Kusano KF Wollins J Welt F Shah P Soukas P Asahara T Henry TD Intramyocardial transplantation of autologous CD34+ stem cells for intractable angina: a phase I/IIa double-blind, randomized controlled trial Circulation 2007 115 25 3165 72 10.1161/CIRCULATIONAHA.106.687376 17562958

[B14] Pompilio G Steinhoff G Liebold A Pesce M Alamanni F Capogrossi MC Biglioli P Direct minimally invasive intramyocardial injection of bone marrow-derived AC133+ stem cells in patients with refractory ischemia: preliminary results Thorac Cardiovasc Surg 2008 56 2 71 6 10.1055/s-2007-989351 18278680

[B15] Horvath KA Chiu E Maun DC Lomasney JW Greene R Pearce WH Fullerton DA Up-regulation of vascular endothelial growth factor mRNA and angiogenesis after transmyocardial laser revascularization Ann Thorac Surg 1999 68 3 825 9 10.1016/S0003-4975(99)00842-5 10509969

[B16] Hughes GC Kypson AP Annex BH Yin B St Louis JD Biswas SS Coleman RE DeGrado TR Donovan CL Landolfo KP Lowe JE Induction of angiogenesis after TMR: a comparison of holmium: YAG, CO2, and excimer lasers Ann Thorac Surg 2000 70 2 504 9 10.1016/S0003-4975(00)01569-1 10969671

[B17] Hughes GC Biswas SS Yin B Baklanov DV Annex BH Coleman RE DeGrado TR Landolfo CK Landolfo KP Lowe JE A comparison of mechanical and laser transmyocardial revascularization for induction of angiogenesis and arteriogenesis in chronically ischemic myocardium J Am Coll Cardiol 2002 39 7 1220 8 10.1016/S0735-1097(02)01734-5 11923050

[B18] Lutter G Attmann T Heilmann C von Samson P von Specht B Beyersdorf F The combined use of transmyocardial laser revascularization (TMLR) and fibroblastic growth factor (FGF-2) enhances perfusion and regional contractility in chronically ischemic porcine hearts Eur J Cardiothorac Surg 2002 22 5 753 61 10.1016/S1010-7940(02)00527-4 12414042

[B19] Atluri P Panlilio CM Liao GP Suarez EE McCormick RC Hiesinger W Cohen JE Smith MJ Patel AB Feng W Woo YJ Transmyocardial revascularization to enhance myocardial vasculogenesis and hemodynamic function J Thorac Cardiovasc Surg 2008 135 2 283 91 291 e1; discussion 291 Epub 2008 Jan 11. 10.1016/j.jtcvs.2007.09.043 18242252

[B20] Patel AN Spadaccio C Kuzman M Park E Fischer DW Stice SL Mullangi C Toma C Improved cell survival in infarcted myocardium using a novel combination transmyocardial laser and cell delivery system Cell Transplant 2007 16 9 899 905 10.3727/096368907783338253 18293888

[B21] Klein HM Ghodsizad A Borowski A Saleh A Draganov J Poll L Stoldt V Feifel N Piecharczek C Burchardt ER Stockschläder M Gams E Autologous bone marrow-derived stem cell therapy in combination with TMLR. A novel therapeutic option for endstage coronary heart disease: report on 2 cases Heart Surg Forum 2004 7 5 E416 9 10.1532/HSF98.20041095 15799915

[B22] Gowdak LH Schettert IT Rochitte CE Lisboa LA Dallan LA Cesar LA Krieger JE Ramires JA Oliveira SA Cell therapy plus transmyocardial laser revascularization for refractory angina Ann Thorac Surg 2005 80 2 712 4 10.1016/j.athoracsur.2005.04.080 16039237

[B23] Heidt MC Sedding D Stracke SK Stadlbauer T Boening A Vogt PR Schönburg M Measurement of myocardial oxigen tension: a valid and sensitive method in the investigation of transmyocardial laser revascularization in an acute ischemia model Thorac Cardiovasc Surg 2009 57 2 79 84 10.1055/s-2008-1039209 19241308

[B24] Abdel-Latif A Bolli R Tleyjeh IM Montori VM Perin EC Hornung CA Zuba-Surma EK Al-Mallah M Dawn B Adult bone marrow-derived cells for cardiac repair: a systematic review and meta-analysis Arch Intern Med 2007 167 10 989 97 10.1001/archinte.167.10.989 17533201

